# A scoping review of assessment of front-of-pack labeling policy implementation and response in low-and middle-income countries

**DOI:** 10.1017/S136898002510150X

**Published:** 2025-12-04

**Authors:** Payao Phonsuk, Penny Farrell, Kavita Chinoy, Jintana Jankhotkaew, Anne Marie Thow, Sirinya Phulkerd

**Affiliations:** 1 Leeder Centre for Health Policy, Economics & Data, Faculty of Medicine and Health, The University of Sydney, Sydney, NSW, Australia; 2 Department of Health Education and Behavioural Sciences, Faculty of Public Health, Mahidol University, Bangkok, Thailand; 3 School of Public Health, Faculty of Medicine and Health, The University of Sydney, Sydney, NSW, Australia; 4 International Health Policy Program, Nonthaburi, Thailand; 5 Institute for Population and Social Research, Mahidol University, Nakhon Pathom, Thailand

**Keywords:** Front-of pack labeling, Policy implementation, Policy response, Low- and middle-income countries, Scoping review

## Abstract

**Objective:**

Despite growing front-of-pack labelling (FOPL) policy implementation in low-and middle-income countries (LMIC), research approaches for evaluating these policies remain poorly characterized, hindering evidence-based policy development and methodological gaps. This study explored research approaches, frameworks, and methods used in assessing FOPL policy implementation and response in LMIC.

**Design:**

Systematic search of five databases, including Medline, Web of Science, Scopus, Global Health, and CINAHL, for peer-reviewed articles published between 2014–2025. Studies on FOPL policy implementation or response in LMIC were included. Data on study characteristics, methods, and findings were extracted and synthesized.

**Setting:**

LMIC.

**Participants:**

All populations.

**Results:**

Thirty-one studies revealed significant research imbalances. Implementation studies (*n* 3) used qualitative approaches with policy theories, while response studies (*n* 28) predominantly employed quantitative methods including surveys, experiments, and modeling. Pronounced geographical bias emerged, with 24 studies conducted in Latin America while other LMIC regions remained underrepresented. Common limitations included non-representative sampling, self-reported data, and short timeframes. Mandatory FOPL policies achieved higher compliance than voluntary schemes, though implementation faced challenges including inadequate monitoring, limited resources, and industry resistance. Consumer awareness was generally high but varied significantly across population groups, revealing substantial equity gaps.

**Conclusions:**

This review reveals critical gaps in FOPL implementation research in LMIC, with evidence heavily skewed toward consumer responses and geographically concentrated in Latin America. Future research should prioritize implementation science approaches, geographical diversity, and understanding policy processes in resource-constrained settings to develop effective, context-appropriate FOPL policies.

Non-communicable diseases (NCDs) such as CVD, diabetes, and cancer represent a substantial global health burden and contribute significantly to mortality, with unhealthy dietary patterns being a major risk factor^(1)^. Excessive consumption of processed foods and insufficient intake of fruits and vegetables contribute significantly to this epidemic^(2)^. To address these dietary risk factors, front-of-pack labelling (FOPL) has been introduced by the World Health Organization (WHO) as a ‘best-buy’ NCDs prevention intervention^(3)^, providing consumers with clear and accessible nutrition information at the point of purchase^(4–6)^.

Various forms of FOPL, including interpretive and non-interpretive labels have been developed to present critical nutrition information^(7)^. Interpretive labels (such as traffic light labels and warning labels) simplify nutritional information using symbols or color codes to help consumers quickly assess a product’s healthiness, while non-interpretive labels (such as Guideline Daily Amount (GDA)) present factual nutritional information without evaluative guidance^(7)^. Systematic reviews show interpretive labels are generally more effective in improving consumer understanding and influencing purchasing decisions^(8–10)^, though their impact in real-world settings has been variable. The Nutri-Score system, voluntarily adopted in various countries in Europe such as France, Spain, Belgium, and Germany, has boosted the purchase of foods with better nutritional quality but showed no noticeable effect on the purchase of foods with moderate, low, or unlabeled nutritional quality^(11)^. The warning labels, adopted in Chile in 2016, were associated with a reduction in unhealthy food purchasing in the first phase of implementation^(12)^. In contrast, non-interpretive labels like GDA have shown limited effectiveness in changing Britain consumer behavior, often due to difficulties in interpretation^(13)^, although they do increase consumer awareness of nutritional information^(14)^ among European populations.

## Low- and middle-income countries-specific considerations in front-of-pack labelling research

Policy implementation is crucial yet challenging, particularly at the national level. Although the WHO has advocated for the use of FOPL to promote healthier diets since 2004^(15)^, there is limited standardized guidance on how best to develop and implement FOPL, and which stakeholders to involve in its enforcement^(7,15)^. From a policy perspective, significant challenges exist in managing conflicts of interest and industry interference^(7)^.

While some countries have successfully implemented FOPL systems with positive impacts on consumer behavior^(16,17)^ and product reformulation^(18,19)^, significant gaps remain in understanding FOPL effectiveness in low- and middle-income countries (LMIC) contexts. LMIC often face significant constraints in regulatory capacity, limited resources for monitoring and enforcement, and competing policy priorities^(15,20)^. These implementation challenges require research approaches that can capture policy processes operating under resource constraints and examine how limited enforcement capacity affects industry compliance and consumer response patterns.

LMIC food markets typically have different structures, with greater presence of local and regional producers alongside multinational corporations, varying technical capacities for product reformulation, and different economic pressures. Industry response patterns in LMIC may involve distinct strategies that require context-specific research methodologies to understand compliance mechanisms and reformulation capabilities^(21)^.

The nutrition landscape in LMIC presents unique contextual factors that affect FOPL policy effectiveness. Many LMIC are experiencing rapid nutrition transitions with simultaneous exposure to traditional and processed food systems^(22)^, creating complex food environments that differ markedly from established markets in high-income countries. Additionally, there is limited evidence on how diverse socio-economic and cultural factors in LMIC influence FOPL interpretation and use^(23)^, including variations in literacy levels, cultural interpretations of health information, and economic constraints on food choices.

These LMIC-specific factors collectively demonstrate why research methodologies developed in high-income countries cannot be directly extrapolated to LMIC contexts. Developing context-appropriate research approaches for FOPL policy evaluation in LMIC is therefore essential for informing effective policies in settings where most of the world’s population resides.

## Knowledge gaps and research needs

Despite growing FOPL policy adoption across LMIC, there is limited understanding of the research approaches and methodologies used to evaluate these policies. While studies have examined consumer responses to FOPL, there is a paucity of research on the challenges and best practices in policy implementation processes in resource-constrained settings^(15,20)^. Moreover, the theoretical frameworks, measurement tools, and analytical methods used in LMIC contexts remain poorly characterized, hindering the development of evidence-based policy recommendations appropriate for these settings. Understanding these dynamics is crucial for developing context-appropriate policies and ensuring their effectiveness in improving population health across diverse global settings.

This study aimed to (1) explore research approaches, theoretical frameworks, tools, and data analysis methods used in assessing FOPL policy implementation and response in LMIC, and (2) identify methodological gaps and patterns in this literature.

## Conceptual framework for front-of-pack labelling policy implementation and response

**Figure 1:**
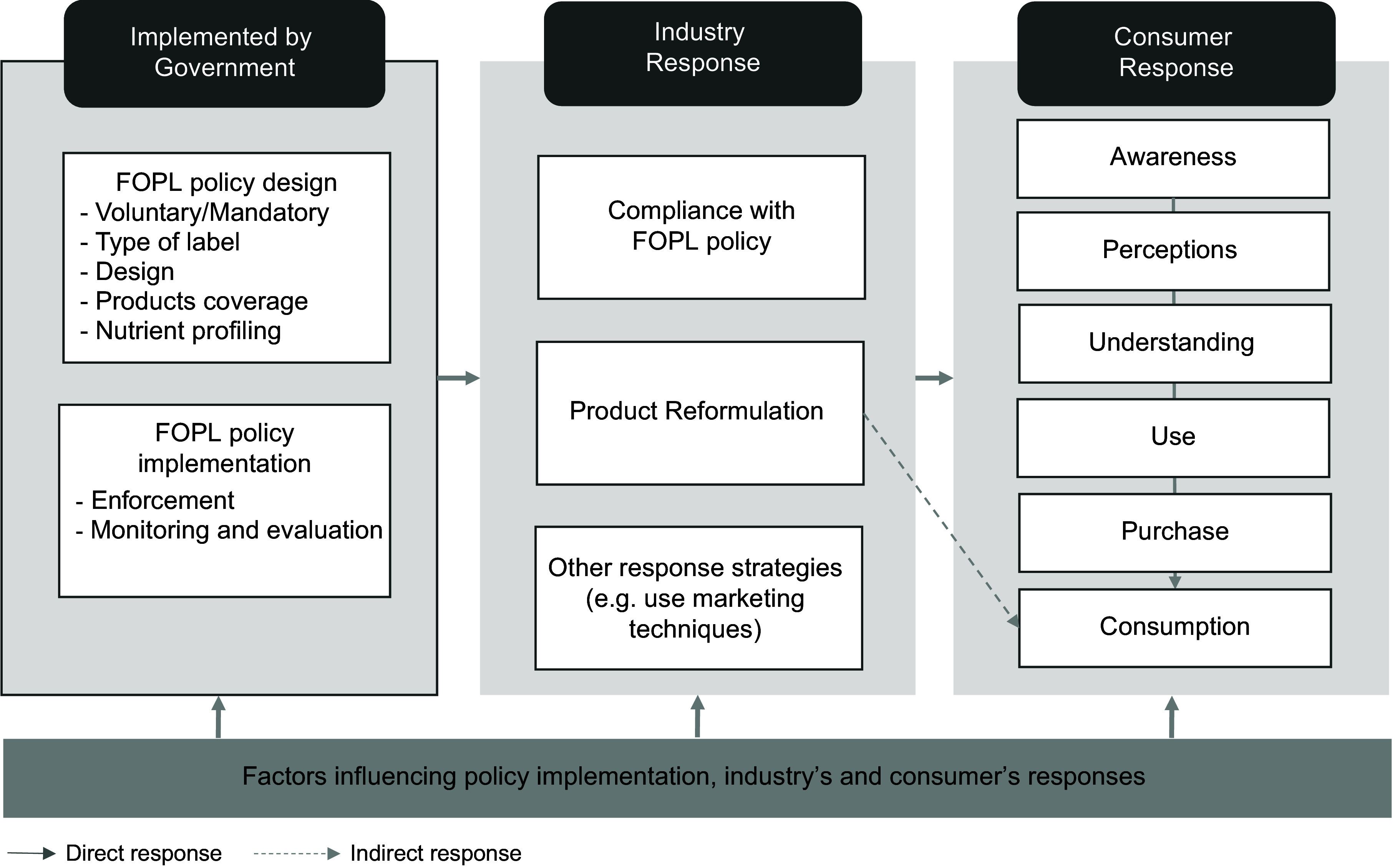
Conceptual framework of front-of-pack labelling (FOPL) policy implementation and response.

Understanding FOPL effectiveness requires examining the complex interactions between policy design, implementation processes, and stakeholder responses. Drawing from Grunert and Wills^(24)^, WHO’s FOPL policy implementation guidelines^(25)^, and the World Cancer Research Fund report on the FOPL policy implementation lessons^(26)^, we developed a conceptual framework that examines how stakeholder dynamics, political context, and industry responses shape policy outcomes across different stages (Figure 1).

This framework identifies three primary actors in FOPL policy processes: government, industry, and consumers, each operating within specific implementation and response pathways. The national government undertakes the design of the FOPL policy, determining label types, format, and product coverage^(25)^, establishes nutrient profiling criteria^(27)^, and manages implementation approaches (voluntary or mandatory) with accompanying monitoring, evaluation, and enforcement mechanisms^(25)^. Industry response primarily involves compliance and reformulation. Compliance requires changes in packaging design and production processes, varying by implementation approach (voluntary or mandatory) and enforcement mechanisms^(25)^. Product reformulation may involve reducing nutrients of concern or increasing beneficial nutrients^(18,28–30)^, influenced by technical feasibility, costs, and consumer acceptance^(31)^. Furthermore, the industry may adopt other strategies such as marketing to maintain their profit margins^(22,32)^. Consumer response occurs through several potential pathways^(24,25)^ including awareness of FOPL presence, understanding of label information, and utilization through informed food choices and purchasing behavior^(13)^. The implementation and response of FOPL policies are also influenced by a range of contextual factors. These factors include contextual elements such as personal knowledge, food availability, public health campaigns, and socioeconomic variables, which can affect the effectiveness and impact of the FOPL policy^(33,34)^.

## Methods

### Search strategies

The method for conducting this review followed the scoping review protocol outlined by the Joanna Briggs Institute^(35)^ and the Preferred Reporting Items for Systematic Reviews and Meta-Analyses extension for Scoping Reviews (PRISMA-ScR) checklist^(36)^ (see online supplementary material, Supplemental Table S1). Peer-reviewed journal articles were selected from five databases: Medline, Web of Science, Scopus, Global Health and CINAHL, supported by the University of Sydney Library. A search strategy was developed in the Medline database and revised accordingly for other databases. Key search terms included ‘front-of-pack’, ‘labelling’, ‘food’, ‘nutrition’, ‘policy’, and ‘implementation’. The details of search strategy and search terms are provided in online supplementary material, Supplemental Table S2. All searches were initially conducted on 9 November 2023 and subsequently updated on 20 August 2025.

### Data collection and analysis

We included only original articles and primary research studies, conducted in LMIC. The focus was on studies that investigate FOPL policies, in any label format, implemented at the national level regardless of their policy mandates and study design. To be included, studies must: (1) be peer-reviewed papers with full-text accessible, (2) be published in English between 2014 and 2025, (3) assess and analyze FOPL policy implementation and response, and (4) employ research approaches, theories, frameworks, tools, or measurements to evaluate and analyze FOPL policy implementation or responses. The policy responses were defined by the conceptual framework, and included product reformulation, consumer awareness, perception, understanding and use of FOPL. The responses were extended to purchasing and consumption behaviors, and others if relevant. Papers were excluded when focused on the impact of FOPL on health and economic outcomes, to maintain focus on methodological approaches to studying implementation and response processes rather than ultimate health impacts, which would require different methodological considerations.

All papers identified through searches in five databases were imported into Covidence software^(37)^. Duplicate entries were removed. Title and abstract screening run by PP and JJ independently. Relevant data on FOPL policy (policy mandate, type of label, product coverage, and year of policy Implementation), study design (study type, populations, setting, tools and measurement, data source and collection, and data analysis), study outcomes (key findings), and limitations and future research mentioned in the papers were extracted by two researchers (PP and KC) into Excel. Conflicts during data screening and extraction were discussed, and a consensus was reached between the researchers. Detailed critical appraisal of the research study design was not included. Following the Joanna Briggs Institute Methodology for Scoping Reviews^(38)^, detailed critical appraisal of the research study design was not conducted as this is not a required component of scoping review methodology, which focuses on mapping the available evidence rather than assessing its methodological quality.

## Results

### Study characteristics

**Figure 2:**
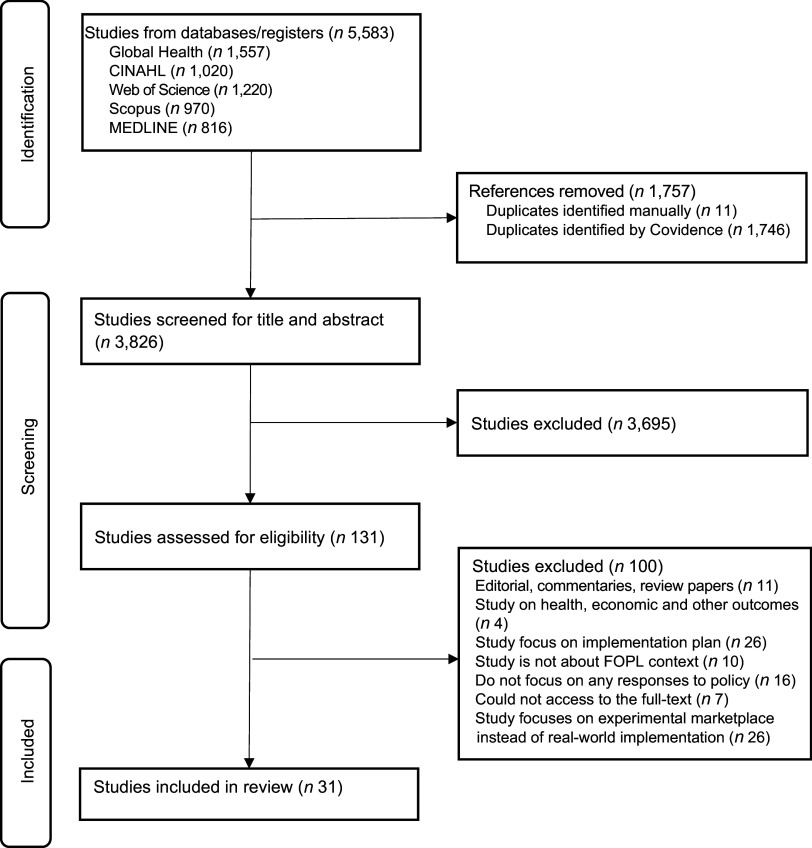
Covidence flow diagram of the paper screening process.

The database search yielded a total of 5583 records for identification. Following the removal of duplicates, 3826 unique papers were screened, of which 131 were selected for full-text review. Ultimately, 31 papers met the inclusion criteria and were included in the analysis (Figure 2). Among these, nine studies were conducted in Mexico, five were done in Brazil. Ecuador and Peru each contributed four studies. Three studies were performed in Thai context. Columbia and Malaysia had each two studies. The remaining studies originated from Iran and Sri Lanka (Table 1).

**Table 1:** Study characteristics

Author and year	Country	Type of label		Policy mandate (Mandatory = M, Voluntary = V)	Year of policy Implementation	Study design	Policy stage focused		Expected outcomes
		Interpretive	Non-interpretive				Implementation	Response	
1. de Alcantara *et al.*, 2025^(39)^	Brazil	Magnifying glass		M	2022	Quantitative (Experiment)		Consumers	Perceived healthfulness of products
2. França *et al.*, 2025^(40)^	Brazil	Magnifying glass		M	2022	Quantitative		Consumers	Perceived healthfulness of products
3. Soares *et al.*, 2025^(41)^	Brazil	Magnifying glass		M	2022	Quantitative (Experiment)		Consumers	Purchasing
4. Senda *et al.*, 2024^(42)^	Brazil	Magnifying glass		M	2022	Quantitative		Industry	Regulation compliance
5. Faria *et al.*, 2023^(43)^	Brazil	Magnifying glass		M	2022	Quantitative (Modelling)		Consumers	Consumption
6. Arboleda, 2025^(44)^	Columbia	Warning Labels		M	2021, revised in 2024	Quantitative (Experiment)		Consumers	Perception on label design and perceived healthfulness of product
7. Rangel-Quinonez *et al.*, 2025^(45)^	Columbia	Warning Labels		M	2021, revised in 2024	Quantitative (Experiment)		Consumers	Purchasing
8. Cordero-Ahiman *et al.*, 2022^(46)^	Ecuador	Traffic Light Label		M	2013	Quantitative		Consumers	Understanding
9. Sandoval *et al.*, 2019^(47)^	Ecuador	Traffic Light Label		M	2014	Quantitative		Consumers	Purchasing
10. *et al.*, 2018^(48)^	Ecuador	Traffic Light Label		M	2014	2 stages; an exploratory study and a quantitative study		Consumers	Emotions
11. Orozco *et al.*, 2017^(49)^	Ecuador	Traffic Light Label		M	2014	Quantitative		Consumers	- Awareness - Understanding - Use
12. Roudsari *et al.*, 2021^(50)^	Iran	Traffic Light Label		M	2016	Qualitative		Consumers	- Attitude - Awareness - Knowledge - Perceptions
13. Sulong *et al.*, 2023^(51)^	Malaysia	Healthier Choice Logo		V	2017	Quantitative		Industry	- Acceptance - Effectiveness of policy in producing healthier products - Reformulation
14. Sulong *et al.*, 2019^(52)^	Malaysia	FOPL for Energy icon		M	2012	Quantitative		Consumers	- Awareness - Understanding
15. Salgado *et al.*, 2025^(53)^	Mexico	Warning Labels		M	2020	Quantitative		Industry	Reformulation
16. Contreras-Manzano *et al.*, 2024^(54)^	Mexico	Warning Labels		M	2020	Quantitative		Consumers	Purchasing
17. Arellano-Gómez *et al.*, 2023^(55)^	Mexico	Warning Labels		M	2020	Quantitative		Consumers	Awareness
18. Crosbie *et al.*, 2023^(56)^	Mexico	Warning Labels		M	2020	Qualitative (secondary data analysis)	Stakeholder views		Challenge in implementation
19. Villaverde *et al.*, 2023^(57)^	Mexico	Warning Labels		M	2020	Quantitative		Consumers	Consumption
20. Campos-Nonato *et al.*, 2022^(58)^	Mexico	Warning Labels	GDA	M (both labels)	2020	Quantitative		Consumers	- Perception - Understanding
21. Contreras-Manzano *et al.*, 2022^(59)^	Mexico	Warning Labels		M	2020	Randomized experiment		Consumers	Understanding
22. Sagaceta-Mejía *et al.*, 2022^(60)^	Mexico	Warning Labels	GDA	M (both labels)	2014 (GDA), 2020 (Warning labels)	Quantitative		Consumers	Understanding
23. Nieto *et al.*, 2020^(61)^	Mexico	Nutritional Stamp labels	GDA, Nutrition Facts Table	M (GDA, Nutrition Facts Table) V (Nutritional Stamp labels)	2014 (GDA)	Qualitative		Consumers	- Perception - Understanding
24. Mamani-Urrutia *et al.*, 2025^(62)^	Peru	Warning Labels		M	2019 (1st phase), 2021 (2nd phase)	Quantitative		Industry	Nutritional content
25. Diez-Canseco *et al.*, 2024^(63)^	Peru	Warning Labels		M	2019 (1st phase), 2021 (2nd phase)	Quantitative		Consumers	- Awareness - Understanding - Perception - Use
26. Saavedra-Garcia *et al.*, 2023^(64)^	Peru	Warning Labels		M	2019 (1st phase), 2021 (2nd phase)	Quantitative		Industry	Reformulation
27. Saavedra-Garcia *et al.*, 2022^(65)^	Peru	Warning Labels		M	2019 (1st phase), 2021 (2nd phase)	Quantitative		Industry	Marketing techniques
28. Madurawala *et al.*, 2023^(66)^	Sri Lanka	Traffic Light Label		M	2016 (for sugar-sweetened beverage), 2019 (expanded to solid foods)	Qualitative (interview and documentary reviews)	Stakeholder views		Ideas, institution, and power dynamics influence policy formation and implementation
29. Nguyen Ngoc *et al.*, 2025^(67)^	Thailand	Healthier Choice Logo		V	2016	Quantitative		Industry	Nutrition composition in products
30. Nguyen Ngoc *et al.*, 2023^(68)^	Thailand	Healthier Choice Logo		V	2016	Quantitative		Industry	Uptake of products
31. Phulkerd *et al.*, 2017^(69)^	Thailand	25 % SFS (products that reduced sugar, fat and sodium by 25 %)		V	2009	Qualitative	Stakeholder views		Barriers and facilitators to policy implementation

The timeline of FOPL policy implementation varies across countries. Brazil introduced its Magnifying Glass label in 2022^(39–43)^. Columbia adopted the warning labels as circular black label in 2021, and the label was changed into octagonal ones and added warnings for trans fats and sweeteners, and eliminated the optional positive label in 2024^(44,45)^. Ecuador adopted the Traffic Light label in 2013^(46–49)^, followed by Iran in 2016^(50)^. Malaysia implemented two types of labels: the voluntary Healthier Choice Logo (HCL) in 2017^(51)^ and the mandatory Energy Icon label in 2012^(52)^. Mexico had several milestones, with a national FOPL law introduced in October 2020, though earlier studies, such as one from September 2019, reflect pre-implementation considerations. Mexico also implemented the warning labels in 2020^(53–60)^, after the implemented the GDA labels in 2014^(60,61)^. Peru’s warning labels policy was rolled out in two phases, starting in 2019–2020, with the second phase in 2021^(62–65)^. Sri Lanka developed the Traffic Light label in 2016 for sugar-sweetened beverage and the label was expanded to solid foods in 2019^(66)^. Thailand had a voluntary label HCL in 2016^(67,68)^ which happened after a 25 % reduction in sugar, fat, and sodium (25 % SFS)^(69)^ was implemented in 2009.

Interpretive labels were commonly used across all countries such as the warning labels in most Latin American countries and the HCL in Malaysia and Thailand. Non-interpretive were also observed including GDA. Most FOPL policies were government-mandated, with mandatory labels such as the Magnifying Glass in Brazil, the Traffic Light label in Ecuador, Iran and Sri Lanka, and the warning labels in Columbia, Mexico and Peru. Voluntary labels were implemented, including Malaysia’s HCL, Mexico’s Nutritional Stamp, and Thailand’s HCL and 25 % SFS.

### Studies focusing on policy implementation

#### Research approaches

Three out of thirty-one studies primarily examined the implementation of FOPL policies, including one each study from Mexico and Sri Lanka, and Thailand. A qualitative approach focusing on secondary data analysis and semi-structured in-depth interviews was employed as main study design. Data collection tools ranged from media reports and legislative reviews to specialized product databases and interviewed guided by theory, all contributing to a detailed examination of the factors affecting the success of food labelling policies.

A qualitative secondary data analysis conducted in Mexico^(56)^ focused on the role of key stakeholders in implementing warning labels. This study used secondary data obtained from various online sources, including media reports, government websites, and legislative documents. The study applied the policy cycle model to explore the factors influencing both the implementation and responses to the policy. Sri Lanka’s study^(66)^ employed interviews and documentary analysis to explore how ideas, institutions, and power dynamics influence the formulation and implementation of the policy. A study in Thailand^(69)^ employed a qualitative study design to identify the barriers and facilitators to implementing a 25 % SFS. This study was based on a qualitative approach using in-depth interviews among a broad range of stakeholders, such as government entities, NGOs, academics, private sectors, and multisector organizations.

These studies applied a theoretical framework to analyze FOPL policy implementation. The Mexican study applied Knill and Tosun’s policy cycle model to examine implementation standards, monitoring and enforcement mechanisms, and policy evaluation^(56)^. The Sri Lankan study drew upon Kingdon’s theory of agenda-setting and Campbell’s institutionalist approach to Political Economy Analysis to examine the policy development process and institutional factors influencing FOPL implementation^(66)^. The Thai study synthesized three categories of theories (classic behavioral, implementation, and adapted frameworks) to develop interview themes across four domains: policy characteristics, individual adopter characteristics, intra-organization characteristics, and environmental influences^(69)^.

All studies exhibited limitations: the secondary data analysis lacked comprehensive review of public comments and in-depth examination of governmental interests^(56,69)^, while the Thai study was constrained by the absence of successful policy implementation examples^(69)^. Additional constraints included limited industry representation, particularly from carbonated beverage manufacturers, and documentation gaps in government institutes that prevented access to relevant policy documents during initial screening stages^(66)^.

#### Key findings from the study on the front-of-pack labelling policy implementation

Studies from Mexico, Sri Lanka and Thailand reveal consistent FOPL implementation challenges. Mexico’s warning labels faced enforcement capacity limitations and regulatory gaps that compromised industry compliance^(56)^. Similarly, the Traffic Light label policies in Sri Lanka aimed at discouraging SSB consumption faced industry resistance and achieved limited effectiveness compared to taxation due to insufficient public awareness, despite Ministry of Health leadership and industry reformulation responses^(66)^. Thailand’s voluntary 25 % SFS labels encountered monitoring deficiencies and insufficient funding allocation but benefited from strong governmental support^(69)^.

### Studies focused on policy response

#### Research approaches

There were 28 studies focused on the response to the FOPL policies. Among those, eight studies focused on the response from the industry, mainly on reformulation efforts and compliance with labelling regulations^(42,51,53,62,64,65,67,68)^. The remaining 20 studies explored consumer responses, awareness and perception of FOPL^(39,40,44,48–50,52,55,58,61,63)^, understanding and use of FOPL^(46,49,52,58–61,63)^, and changes in purchasing behavior^(41,45,47,54)^ and consumption^(43,57)^. This distribution highlights a research emphasis on understanding the impact of FOPL policies, particularly from the perspective of consumers.

The studies employed a range of methodologies to examine the FOPL policy responses across different countries. Quantitative approaches were predominant. These included, for example, cross-sectional surveys and questionnaires to assess understanding of FOPL^(46,49,52,58)^, experimental designs comparing different label types or scenarios^(39,41,44,45,55,59)^, and modelling studies estimating potential impacts on consumption and health outcomes^(43)^. Qualitative methods, including focus groups and in-depth interviews, were used in three studies to explore perceptions, attitudes, and understanding of FOPL^(50,61,63)^. Data sources varied, with 16 studies collecting primary data through surveys, experiments, and direct observation of products^(39–42,44–46,48–52,58,59,61,65)^, while 9 studies utilized secondary data from national health surveys, national panel data, and market research databases such as Euromonitor and Kantar World Panel^(43,47,53,55,57,60,64,68)^. The populations studied were diverse, including general adults in most of the studies and specific demographic groups such as women^(48,49)^ and children^(59)^. Sampling methods varied from convenience and purposive sampling in smaller-scale studies to random sampling from national surveys in larger studies. This methodological diversity allowed for a comprehensive examination of FOPL systems, their implementation, and their effects on consumer behavior and product reformulation across different contexts and populations, primarily in Latin American countries (Brazil, Columbia, Ecuador, Mexico, and Peru).

Several limitations on the study design were consistently reported across these studies. Many studies acknowledged limited geographical scope^(50)^, group of participants (only women or mothers)^(49,63)^, or sample sizes^(44,48,54,55,59,63,65)^, potentially affecting the generalizability of results to national populations. Additionally, the focus on urban populations limited the generalizability of the results to rural or lower-income areas, where access to labeled products and consumer understanding of labels may differ^(46,61)^. Self-reported data in some studies introduced potential measurement errors^(51,57)^. The short-term nature of some studies also limited their ability to assess the long-term impacts of FOPL implementation^(58)^. Another limitation was product coverage including restricted sample sizes, focus on specific product categories (e.g. carbonated soft drinks), exclusion of products not available at studied points of sale, and omission of away-from-home consumption^(47,64,65)^.

#### Responding towards front-of-pack labelling policy

Industry response to FOPL policies was evident in compliance of FOPL policy, product reformulation, product uptake and nutrition composition, and marketing strategies^(42,51,53,62,64,65,67,68)^. For example, a study in Brazil showed 541 out of 2145 products displayed warnings label with significant variation across food categories^(42)^. In Mexico, all food groups reduced their nutrients, with the most significant decreases in products exceeding cutoffs for sodium (up to –63·1 percentage points), saturated fat (up to –26·3 percentage points), and non-caloric sweeteners (up to –29·0 percentage points)^(53)^. Product reformulation mainly occurred post-implementation rather than proactively^(53)^. Peruvian study showed significant nutrient reductions following the warning labels implementation, with total decreases of 3·4 % in calories, 14 % in sodium, 36·7 % in sugar, and 9·2 % in saturated fats^(62)^. In Thailand, a longitudinal study showed a gradual increase in HCL product uptake over five years, with the logo appearing on 10·7 % of total products and 39·5 % of eligible products, though only 19 % of manufacturers (primarily SMEs) launched healthier products with the logo^(68)^. Beverages carrying the HCL exhibited significantly better healthfulness compared to those without the label^(67)^.

Consumer response to FOPL was multifaceted, encompassing awareness, understanding, and behavioral changes. Awareness of FOPL was generally high across studies, ranging from 85 % in Malaysia^(52)^ to 97 % in Ecuador^(46)^. However, awareness varied significantly among different population groups, with one study in Ecuador reporting that 84·3 % of indigenous women were unaware of the labeling system compared to 46 % of mestiza women^(49)^. Furthermore, warning labels consistently demonstrated superior comprehension compared to GDA labels^(58)^.

The impact of FOPL on purchasing behavior and consumption showed mixed results. Studies in Brazil, Columbia and Mexico reported a reduction on purchasing high-energy and high-sugar products with the benefit from interpretive labels (magnifying glass and warning labels)^(41,45,54)^. Additionally, modeling studies in Brazil and Mexico projected significant potential reductions in energy and nutrient intake, including a 54·1 % decrease in added sugar consumption^(43,57)^. However, no definitive evidence was found that Traffic Light Nutritional Labeling reduced purchases of carbonated soft drinks in Ecuador^(47)^.

The review found that responses to FOPL varied significantly across different population groups. Children showed distinct patterns of label comprehension, with a study in Mexico finding that warning labels led to a higher percentage of children correctly identifying the healthiest and least healthy options compared to Nutrient Fact panels^(59)^. Among adults, those with NCDs showed better understanding of warning labels compared to GDA labels^(60)^. Socioeconomic status also influenced responses, with higher-status households in Ecuador tending to purchase less high-sugar and more low- and non-sugar soft drinks^(47)^.

### Cross-cutting factors influencing implementation and response

Several factors influence FOPL policy effectiveness across personal, environmental, and policy levels. At the personal level, socio-demographic characteristics emerge as significant determinants. Age plays an influential role, with children showing distinct patterns of label comprehension^(59)^, while educational levels positively correlate with better understanding and use of labels^(45,58,60)^. Socioeconomic status is another crucial factor, with higher-status households more likely to purchase lower-sugar products in response to labeling^(47)^. Health status also impacts FOPL effectiveness, as individuals with NCDs demonstrate improved comprehension of warning labels compared to other label types^(60)^. Emotional responses, though less studied, influence consumer perceptions, as seen in Ecuador where red labels in traffic light systems elicited more fear and guilt, particularly among lower-income consumers^(48)^.

From an industry and policy perspective, company size may affect the adoption of healthier product logos, with small and medium enterprises being more likely to launch new products with such logos^(51)^. The design and implementation approach of FOPL policies themselves influence outcomes, with mandatory approaches generally yielding higher compliance and impact compared to voluntary schemes^(69)^. These findings underscore the complex interplay of personal factors (socio-demographics, health status, emotions), industry characteristics, and policy-level factors in shaping FOPL effectiveness.

## Discussion

This review reveals that FOPL research in LMIC predominantly focuses on policy responses (28 studies) rather than implementation processes (3 studies), highlighting a significant research gap. Among those response studies, there is a notable skew toward consumer responses, with relatively less focus on industry responses. The scarcity of industry-focused studies in FOPL research represents a notable gap in the literature. This includes, for example, an examining of industry compliance mechanisms, challenges, and interference in FOPL policy development in resource-constrained LMIC settings, where industry has been found to actively influence policies through various strategies including direct opposition to mandatory schemes, proposing alternative labeling systems, and questioning the evidence base^(21)^. Understanding these industry dynamics is crucial for designing effective and feasible FOPL policies that can achieve public health objectives while considering industry capabilities.

The implementation of FOPL policies has catalyzed a complex interplay between government regulations and industry responses. Our review reveals distinct patterns between mandatory and voluntary policy approaches. Mandatory approaches demonstrate higher compliance and greater potential impact compared to voluntary schemes^(70)^, as evidenced by formal compliance through product reformulation under mandatory schemes, such as Peru’s reduction in sugar content^(64)^. In contrast, voluntary approaches allow industry greater autonomy in their responses, often resulting in strategic adaptations like increased marketing efforts rather than substantive product changes^(65)^. This dynamic illustrates the fundamental relationship between regulatory policy and industry behavior, where industry actively advocates for voluntary approaches while influencing enforcement strategies through arguments about market efficiency and economic feasibility^(71)^. The clearer compliance patterns observed under mandatory schemes, compared to voluntary approaches, underscore how different policy approaches fundamentally shape industry responses and ultimately affect policy effectiveness^(7)^.

FOPL policy responses are observed through product reformulation, which presents both opportunities and limitations. On the positive side, reformulation can create population-wide reductions in nutrient intake without requiring individual behavior change^(72)^. Studies of reformulation programs have shown modest but meaningful reductions in population fat, salt, and sugar consumption when changes are implemented systematically across product categories^(18,73–75)^. However, several limitations emerge. The nutritional quality of reformulated products often remains questionable, particularly when artificial sweeteners or salt substitutes are used^(76)^. Further, reformulation may create a ‘health halo’ effect^(77)^, potentially encouraging increased consumption of relatively unhealthy products despite marginal improvements^(78)^.

Our review reveals a significant imbalance in the focus of FOPL research, with most studies (28 out of 31) concentrating on responses to policies. This pattern may reflect several underlying factors, particularly in the LMIC context. First, measuring responses, particularly among the consumer group, often requires less complex study designs and can be accomplished through straightforward quantitative methods such as surveys and experimental studies, as seen in Ecuador^(46,49)^ and Malaysia^(52)^. In contrast, studying implementation processes is complex and requires more resource-intensive approaches, including stakeholder interviews, policy document analysis, and long-term monitoring^(79)^, as demonstrated in the Thai study examining implementation barriers^(69)^. Second, accessing implementation data may be more challenging as it often requires engagement with government stakeholders and industry actors^(79)^. Additionally, while consumer response studies can be conducted relatively quickly after policy introduction, meaningful implementation research requires policies to be well-established^(15)^, which may explain the limited implementation studies given the relatively recent adoption of FOPL policies in many LMIC.

The geographical imbalance of included studies limits our policy implication to other LMIC. 24 of 31 studies conducted in Latin American countries (Brazil, Colombia, Ecuador, Mexico, and Peru), while other LMIC regions remain severely underrepresented. Only seven studies originated from other regions: three from Thailand, two from Malaysia, and one each from Iran and Sri Lanka. This geographical bias limits our ability to make broad generalizations^(80)^ about FOPL policy implementation and response across all LMIC, as our findings predominantly reflect Latin American experiences with mandatory warning labels and traffic light systems^(81)^. The limited representation from Africa, South Asia, and most of Southeast Asia represents a critical evidence gap, as these regions may face different regulatory contexts, food market structures, industry dynamics, and cultural factors that influence FOPL effectiveness^(21,82,83)^. Consequently, our conclusions should be interpreted primarily as insights into Latin American FOPL policy experiences rather than universal LMIC patterns.

### Research implications

The patterns identified in this review highlight a fundamental mismatch between research priorities and policy needs in FOPL implementation. While most studies focus on measuring consumer and industry responses to existing policies, there is insufficient understanding of the implementation processes that determine whether policies achieve their intended effects. This research-practice gap is particularly problematic in LMIC contexts, where resource constraints, regulatory capacity limitations, and diverse stakeholder interests create complex implementation challenges that differ markedly from high-income country experiences. The geographical concentration of evidence in Latin America, combined with the predominance of response-focused studies, suggests that current research approaches may not adequately capture the full spectrum of FOPL policy experiences across diverse LMIC contexts.

These findings underscore the importance of ongoing monitoring and adaptive regulation in FOPL policy implementation. Policymakers should anticipate and address potential industry workarounds to maintain the long-term effectiveness of FOPL policies^(84)^. Regulatory frameworks need to be both stringent and flexible, ensuring industry actions align with public health objectives while allowing for a responsive approach to emerging strategies^(7)^.

### Study limitations

The literature search was restricted to English-language peer-reviewed publications, which may have excluded relevant studies from non-English speaking LMIC and valuable implementation insights from government reports and policy documents in grey literature. The timeframe of included studies (2014–2025) may not fully capture complete policy cycles and long-term impacts, particularly for policies adopted early in this period. Additionally, focusing exclusively on LMIC may have missed valuable insights from high-income countries’ FOPL experiences.

### Future research recommendations

To advance FOPL policy effectiveness in LMIC, future research must prioritize several key areas. Most urgently, implementation science approaches should be expanded to balance between response and implementation studies, with particular focus on understanding policy processes in resource-constrained settings. Research programs should systematically investigate industry compliance mechanisms across diverse LMIC market structures, examining how multinational corporations and local producers respond differently to FOPL regulations. Equity-focused studies are essential to address the substantial variations in FOPL effectiveness across population groups, as demonstrated by the 84 % *v*. 46 % awareness gaps between indigenous and non-indigenous women in Ecuador^(49)^. Methodological innovations should include longitudinal designs, representative sampling strategies, and mixed-methods approaches that can capture both immediate responses and long-term policy impacts. Finally, as our review included only English publications, future reviews could benefit from AI-based translation tools to include studies published in other languages, particularly Spanish and Portuguese given the concentration of FOPL policies in Latin America.

## Conclusions

This scoping review reveals a critical research imbalance in FOPL policy evaluation in LMIC, with response-focused studies vastly outnumbering implementation research, and evidence heavily concentrated in Latin America. While findings demonstrate that mandatory FOPL approaches consistently outperform voluntary schemes in achieving compliance and consumer impact, persistent implementation challenges including inadequate monitoring systems, limited regulatory capacity, and industry resistance remain poorly understood due to insufficient implementation science research. The pronounced geographical bias limits generalizability across diverse LMIC contexts, where varying regulatory frameworks, market structures, and cultural factors may significantly influence policy outcomes.

Future research must prioritize implementation science approaches to balance the current portfolio and provide practical guidance for policymakers in resource-constrained settings. Critical needs include geographically diverse studies across underrepresented regions, particularly Africa, South Asia, and Southeast Asia; longitudinal designs to capture policy evolution and sustained impacts; and interdisciplinary research examining how international trade agreements influence FOPL policy adoption. Only through comprehensive, equity-focused research that addresses both methodological and geographical gaps can the full potential of FOPL policies be realized in improving population health across LMIC.

## Supporting information

Phonsuk et al. supplementary material Phonsuk et al. supplementary material

## Supplementary material

For supplementary material accompanying this paper visit https://doi.org/10.1017/S136898002510150X
